# Retraction: Several intuitionistic fuzzy hamy mean operators with complex interval values and their application in assessing the quality of tourism services

**DOI:** 10.1371/journal.pone.0355768

**Published:** 2026-08-11

**Authors:** The PLOS One Editors

The *PLOS One* Editors retract this article [1] due to concerns about potential manipulation of the publication process. These concerns call into question the validity and provenance of the reported results. We regret that the issues were not identified prior to the article’s publication.

ANAK, AA, IM, and MA did not agree with the retraction. MS either did not respond directly or could not be reached.
